# A Transgenic Prox1-Cre-tdTomato Reporter Mouse for Lymphatic Vessel Research

**DOI:** 10.1371/journal.pone.0122976

**Published:** 2015-04-07

**Authors:** Roberta Bianchi, Alvaro Teijeira, Steven T. Proulx, Ailsa J. Christiansen, Catharina D. Seidel, Thomas Rülicke, Taija Mäkinen, René Hägerling, Cornelia Halin, Michael Detmar

**Affiliations:** 1 Institute of Pharmaceutical Sciences, Swiss Federal Institute of Technology, ETH Zurich, Zurich, Switzerland; 2 Institute of Laboratory Animal Sciences, University of Veterinary Medicine, Vienna, Austria; 3 Department of Immunology, Genetics and Pathology, Uppsala University, Uppsala, Sweden; 4 Mammalian Cell Signaling Laboratory, Department of Vascular Cell Biology, Max-Planck Institute for Molecular Biomedicine, Münster, Germany; University of Queensland, AUSTRALIA

## Abstract

The lymphatic vascular system plays an active role in immune cell trafficking, inflammation and cancer spread. In order to provide an *in vivo* tool to improve our understanding of lymphatic vessel function in physiological and pathological conditions, we generated and characterized a tdTomato reporter mouse and crossed it with a mouse line expressing Cre recombinase under the control of the lymphatic specific promoter Prox1 in an inducible fashion. We found that the tdTomato fluorescent signal recapitulates the expression pattern of Prox1 in lymphatic vessels and other known Prox1-expressing organs. Importantly, tdTomato co-localized with the lymphatic markers Prox1, LYVE-1 and podoplanin as assessed by whole-mount immunofluorescence and FACS analysis. The tdTomato reporter was brighter than a previously established red fluorescent reporter line. We confirmed the applicability of this animal model to intravital microscopy of dendritic cell migration into and within lymphatic vessels, and to fluorescence-activated single cell analysis of lymphatic endothelial cells. Additionally, we were able to describe the early morphological changes of the lymphatic vasculature upon induction of skin inflammation. The Prox1-Cre-tdTomato reporter mouse thus shows great potential for lymphatic research.

## Introduction

The lymphatic vascular system has an important physiological role in the maintenance of tissue fluid homeostasis, the transport of antigens and immune cells from the periphery to lymph nodes where the adaptive immune response occurs, and the intestinal absorption of dietary lipids [1]. Moreover, the lymphatic system contributes to a number of pathological processes such as primary and secondary lymphedema, cancer metastasis, inflammation and transplant rejection [2]. In some pathological conditions such as cancer dissemination and transplant rejection, the inhibition of lymphangiogenesis, the growth of new lymphatic vessels (LVs) from pre-existing ones, has been considered as a new therapeutic approach [3]. On the other hand, the activation of lymphangiogenesis might be beneficial for the treatment of lymphedema and chronic skin inflammation [4]. Given the importance of lymphangiogenesis as a therapeutic target and the need for further insights into the contribution of lymphangiogenesis to pathological conditions, substantial efforts have been invested in generating mouse models that allow the visualization of LVs *in vivo* and the isolation of lymphatic endothelial cells (LECs) for transcriptome analyses. To date, several transgenic mouse lines for fluorescent detection of LVs have been described. These lines are based on gene-targeted bacterial artificial chromosome (BAC) transgenic constructs for the expression of either GFP [5], mOrange [6] or tdTomato [7] under *Prox1* transcriptional control. The expression of an EGFP-luciferase dual fluorescent-bioluminescent reporter under the control of *Flt4* (vascular endothelial growth factor 3) regulatory elements has also been reported [8]. Additional LV detection techniques used in mice include positron emission tomography (PET) combined with radiolabeled anti-LYVE-1 antibodies [9], the injection of liposomal preparations of indocyanine green [10] and the use of PEG-conjugated near infrared dyes [11]. Here, we describe the generation of a tdTomato reporter mouse line and show the specific labeling of the LVs after crossing with a Prox1-Cre-ERT2 line [12]. For the first time, we show the applicability of this lymphatic-specific reporter mouse to intravital microscopy (IVM) of dendritic cell (DC) migration and studies of LV morphology during the early phases of cutaneous inflammation, as well as LEC single cell analysis. Our findings indicate that this new mouse model has a great potential for studying the lymphangiogenic process and related functions in physiological and pathological conditions.

## Materials and Methods

### Cloning and in vitro testing of the tdTomato reporter construct

The tdTomato coding sequence was amplified by PCR (forward primer 5’-ATG GTG AGC AAG GGC GAG GA-3’, reverse primer 5’-AAC AAA AGC TGG GTA CCG GGC-3’) and cloned into a pCMVbASIRE construct [13] (kindly provided by Dr. Sabine Werner, ETH Zurich) to obtain the pCMVbASIRE-tdTomato plasmid. The floxed-STOP cassette was excised by transformation of MM294-Cre *E*. *coli* as previously described [14]. Efficient recombination of the STOP cassette was tested by restriction digestion analysis. HEK293 cells were transiently transfected with pCMVbASIRE-tdTomato or the Cre-recombined plasmid using the PEI (polyethylenimine) method and analyzed with an inverted fluorescent microscope (Zeiss) 48 hours after transfection.

### Generation of the lox-STOP-lox (LSL)-tdTomato reporter mouse

pCMVbASIRE-tdTomato was digested with *Acl*I and the 4.8 Kbp fragment (LSL-tdTomato) was purified using the QIAquick gel extraction kit (QIAGEN) and was eluted in sterile water. LSL-tdTomato reporter mice were generated by pronuclear microinjection of the *Acl*I DNA fragment into C57BL/6N-fertilized oocytes. Founders were identified by PCR of genomic DNA using the following primers: FOR 5’-GCG TTA CAT AAC TTA CGG TAA ATG GCC C-3’, REV 5’-GGG CGT ACT TGG CAT ATG ATA CAC TTG ATG-3’. Relative transgene copy number was estimated by real-time PCR on genomic DNA using SYBR green and the following primer pair for the tdTomato transgene: FOR 5’-GCG TTA CAT AAC TTA CGG TAA ATG GCC C-3’, REV 5’-GGG CGT ACT TGG CAT ATG ATA CAC TTG ATG-3’. The following primer pair was used to amplify a control endogenous gene (podoplanin): FOR 5’-AGG GTA TGA AAG CCC CAA GC-3’, REV 5’-GAG ATA CCC AGG GCG AGG TT-3’. Both reactions were found to have the same efficiency; therefore, delta Ct (tdTomato Ct—podoplanin Ct) was used to estimate the relative copy number.

### Mice, breedings and tamoxifen administration

LSL-tdTomato reporter mice were crossed with keratin 5 (K5)-Cre-ERT2 mice obtained from the MMRRC repository (University of Missouri) [15]. Double transgenic animals were identified by genotyping. The back skin of double transgenic animals and wild-type littermates (6–8 weeks old) was shaved and painted with 1 mg 4-hydroxytamoxifen (4-OHT, Sigma) dissolved in ethanol for 5 consecutive days. LSL-tdTomato reporter mice and tdRFP reporter mice [16] were crossed with Prox1-Cre-ERT2 mice [12]. Double transgenic animals were identified by genotyping. Double transgenic adult animals and wild-type control littermates were intraperitoneally injected with tamoxifen three times a week for two weeks (5 l μg/g body weight, 10 mg/ml tamoxifen in sunflower seed oil, Sigma). Prox1-mOrange2 mice were described previously [6].

### Induction of skin inflammation

A contact hypersensitivity reaction to oxazolone was induced in the ears of 7-weeks-old Prox1-Cre-tdTomato mice as described previously [17]. Briefly, a 2% oxazolone solution (4-ethoxymethylene-2 phenyl-2-oxazoline-5-one; Sigma-Aldrich) in acetone/olive oil (4:1 vol/vol) was applied topically to the shaved abdomen (50 μl) and to each paw (5 μl). Five days after sensitization (day 0), both ears were challenged by topical application of 10 μl oxazolone (1%) on each side. Ear thickness was measured with a caliper.

### IVIS imaging, stereomicroscopy and intravital microscopy

K5-Cre-tdTomato mice were imaged using an IVIS spectrum (Caliper Life Sciences, Alameda, CA) with the following settings: excitation 570 nm, emission 620 nm, exposure time: 10 sec, binning: HR4. Prox1-Cre-tdTomato mice were analyzed with a StereoLumar.V12 stereomicroscope (Zeiss) equipped with a 550 nm laser (CoolLED, Andover, UK) and a Texas Red filter set (Zeiss). For intravital confocal and multiphoton microscopy, Prox1-Cre-tdTomato, Prox1-Cre-tdRFP and Prox1-mOrange mice were anesthetized with medetomidine (1 mg/kg) and ketamine (75 mg/kg) or with 3% isofluorane and the ears were depilated with VEET cream. Mice were transferred to a custom-made microscopy stage and placed into a 37°C incubator chamber installed on the microscope platform. Intravital microscopy was performed on a Leica TCS SP8 MP inverted confocal/multiphoton microscope (Leica). Z-Stacks were acquired using a 20x 0.7NA PH2 HC Plan Apochromat objective. Images were analyzed with IMARIS (v7.1.1, Bitplane, Zurich, Switzerland) and ImageJ software.

### Intravital microscopy of dendritic cell migration

Bone marrow derived DCs were generated from the bone marrow of CD11c-YFP mice as described [18]. 5 x 10^5^ bone marrow DCs were injected into the ventral side of the ear pinna. After 4–6 hours, mice were anesthetized with medetomidine (1 mg/kg) and ketamine (75 mg/kg), and the ears were depilated with VEET cream. Intravital microscopy was performed as described by Nitschke et al. [18]. Briefly, mice were transferred to a custom-made microscopy stage and placed into a 37°C incubator chamber installed on the microscope platform. Imaging was performed on a Zeiss LSM 710 inverted confocal microscope (Carl Zeiss AG). A 1 hour time-lapse video including Z-Stacks every 30 seconds was acquired using a 20x 0.8 NA Plan Apochromat objective. An Argon laser (488 nm, for YFP excitation) and a solid-state laser (561 nm, for tdTomato excitation) were used for image acquisition. Videos were analyzed with IMARIS software (v7.1.1, Bitplane, Zurich, Switzerland).

### Ethics statement

All mice used in this study were bred and housed in the animal facility of ETH Zurich. Experiments were performed in accordance with animal protocols 149/2008, 237/2013 and 190/2011 approved by the local veterinary authorities (Kantonales Veterinäramt Zürich).

### Immunofluorescence analysis of tissue whole mounts

Mice were sacrificed, hair was removed with depilation cream and ears were harvested and split into two halves along the cartilage. Ear tissues or lymph nodes were fixed for two hours in 4% PFA at 4° C, washed for 1 hour in PBS and incubated in blocking solution (5% normal donkey serum, 1% BSA, 0.01% Triton-X 100 in PBS) for 4 hours at room temperature. Subsequently, samples were incubated overnight at room temperature with primary antibodies in blocking solution: rabbit anti-RFP (1:300, Rockland), goat anti-LYVE-1 (1:200, R&D Systems), goat anti-Prox1 (1:200, R&D Systems). After extensive washes in PBS, samples were incubated for 2 hours at room temperature with AlexaFluor 488, 594 or 647- conjugated secondary antibodies raised in donkey (1:200, Invitrogen). After at least 2 hours of washes in PBS, samples were mounted with Vectashield mounting media (Vector) on glass slides. Whole mount z-stacks were acquired using an LSM 710 FCS confocal microscope equipped with a 10x 0.3 NA EC Plan-Neofluar objective using ZEN software (Zeiss), and were processed with ImageJ software.

### FACS analysis of ear single cell suspensions

Ears were digested with collagenase IV (Invitrogen) as described [19]. Ear single cell suspensions were stained with the following antibodies: rat anti-CD45-APC-Cy7 (1:250, Biolegend), rat anti-CD31-APC (1:100, BD), hamster anti-podoplanin (1:100, clone 8.1.1, Developmental Studies Hybridoma Bank, University of Iowa) and anti-hamster-Alexa 488 (1:200, Invitrogen). FACS analysis was performed on a BD Fortessa analyzer (BD Biosciences) using the FACSDiva software. Data were analyzed with FlowJo software (TreeStar).

### Ultramicroscopy analysis of lymph node whole mounts

Mice were sacrificed and lymph nodes were dissected and fixed in 4% PFA. After a wash in PBS, samples were permeabilized in 0.5% Triton-X 100 in PBS. After a wash in PBS, samples were blocked in blocking solution (1% BSA, 0.1% Tween-20 in PBS) and subsequently incubated with primary antibodies diluted in blocking solution. The antibodies used were rabbit anti-RFP (1:100, Rockland) and goat anti-LYVE-1 (1:100, R&D Systems). Samples were washed in PBS 0.1% Tween-20 (PBS-T) and incubated with AlexaFluor 488 and 647-conjugated secondary antibodies (1:200, Invitrogen) in blocking solution. After a wash in PBS-T, samples were embedded in 1% ultrapure low melting point agarose, dehydrated with methanol and cleared with BABB (benzyl alcohol/ benzyl benzoate 1:2) [20]. Briefly, samples were dehydrated in a series of 50%, 70%, 95% (at least 1 hour each) and 100% methanol (overnight) and cleared for 1 hour in 50% BABB in methanol and finally in BABB for at least 8 hours. Samples were stored in BABB at 4°C in the dark until image acquisition. Images were acquired using a LaVision Ultramicroscope (LaVision BioTec, Bielefeld). Stacks were captured using 2 μm step size and 2.5x magnification. Maximum projection of a 260 μm z-stack was obtained using the ImageJ software.

## Results

### Generation of the tdTomato reporter mouse

**Fig 1:**
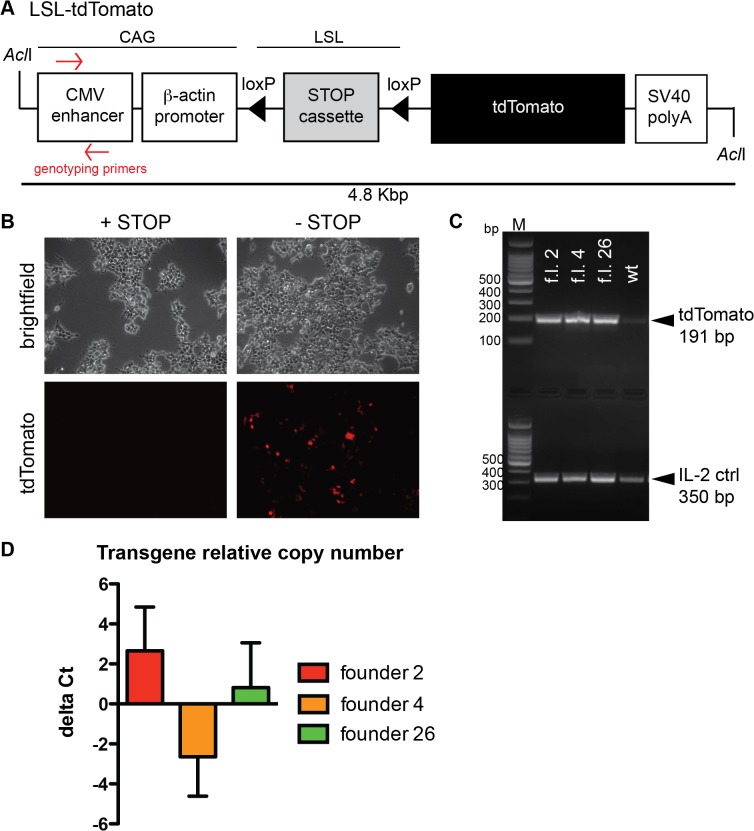
Generation of the tdTomato reporter mouse. **A** Schematic representation of the LSL-tdTomato construct used for pronuclear microinjection. **B** HEK293 cells were transiently transfected with the construct with or without the STOP cassette. TdTomato fluorescence was analyzed 48 hrs post transfection with an inverted microscope and was visible only upon excision of the STOP cassette. **C** PCR genotyping of genomic DNA extracted from ear biopsies identified founder animals (f.l. founder line) positive for the transgene (tdTomato; 191 bp amplicon). A control gene (IL-2; 350 bp amplicon) was used for genomic DNA quality. **D** Transgene relative copy number was estimated by real-time PCR of genomic DNA. Primers specific for the transgene and for a control gene were used and the delta Ct was calculated. Founder 2 carried the least amount of copies, founder 4 the highest and founder 26 an intermediate number.

The tdTomato coding sequence was cloned under the control of a CMV-enhancer, β-actin promoter (CAG) and downstream of a transcriptional/translational-floxed stop cassette (LSL) (Fig 1A), allowing a strong expression of the tdTomato transgene upon Cre recombination of the floxed STOP cassette. In order to test the construct *in vitro*, HEK293 cells were transiently transfected with the plasmid with or without the STOP cassette. TdTomato was expressed specifically upon recombination of the floxed STOP cassette (Fig 1B), confirming that the construct was efficient and not leaky. An *AclI* fragment was utilized for the generation of a transgenic mouse line by injection into the pronucleus of fertilized C57BL/6N oocytes. Five founders were identified by PCR of genomic DNA (Fig 1C) and designated as C57BL/6N-Tg(CAG-tdTomato)581-585Biat. Three founders (number 2, 4 and 26) bred normally and transmitted the transgene to the progeny with Mendelian distribution. The relative copy number of the transgene was estimated by real-time PCR of genomic DNA in comparison with a control gene (podoplanin). Founder 4 carried the highest amount of copies, founder 2 the least and founder 26 an intermediate number of copies (Fig 1D).

### TdTomato is expressed in the skin upon crossing of the LSL-tdTomato reporter mice with a K5-Cre-ERT2 line

**Fig 2:**
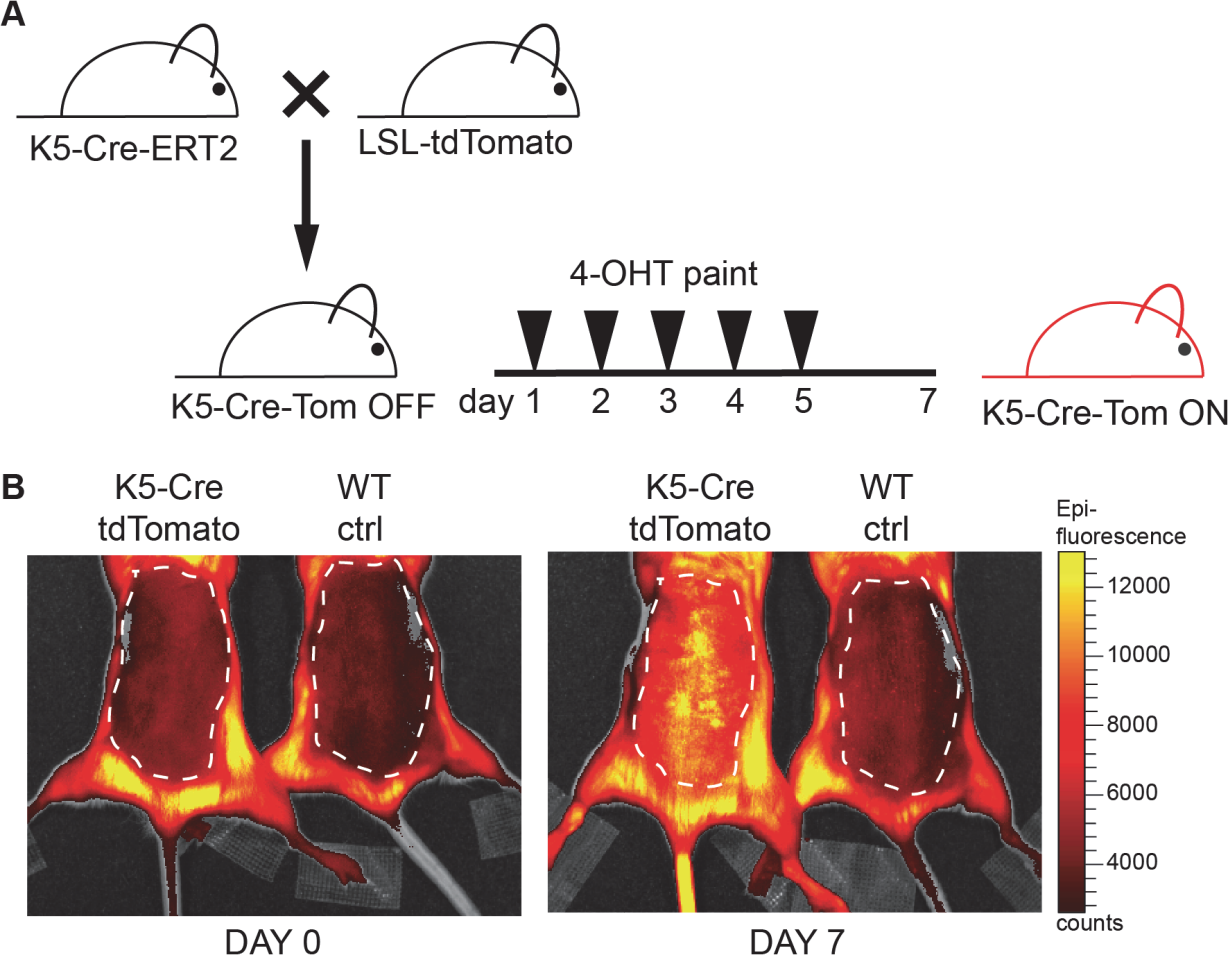
TdTomato is expressed in the skin upon crossing of the reporter mice with a K5-Cre-ERT2 line. **A** Schematic representation of the breeding to K5-Cre-ERT2 and of 4-hydroxytamoxifen (4-OHT) applications on the shaved back skin (1 mg in ethanol for five consecutive days). **B** Prior (day 0) and after 4-OHT application (day 7), mice where imaged with the IVIS spectrum (excitation: 570 nm; emission: 620nm; exposure time: 10 sec; binning: HR4. Color scale min = 2600 counts, max = 13000 counts). TdTomato was expressed in the skin of double positive, treated animals. Dashed line indicates the 4-OHT treated shaved back skin.

To test the expression of tdTomato *in vivo* upon recombination of the STOP cassette, and to select the best founder for further experiments, we crossed the LSL-tdTomato reporter mice with a mouse line expressing Cre recombinase under control of the skin-specific keratin 5 promoter in an inducible fashion (K5-Cre-ERT2) [15]. Cre expression was induced in double transgenic and wild-type littermate adult mice by applying 4-hydroxytamoxifen (4-OHT) in ethanol on the shaved back skin for 5 consecutive days (Fig 2A). Before treatment and two days after the last application, mice were imaged using an IVIS spectrum. Imaging of representative animals derived from founder line 2 clearly demonstrated tdTomato expression in the treated back skin (Fig 2B, dashed line). We also observed systemic activation in other skin compartments (i.e. tail and paws, Fig 2B) since 4-OHT is absorbed through the skin and is readily active. These results confirmed that the LSL-tdTomato reporter mouse is suitable for imaging of tdTomato expression upon genetic recombination *in vivo*. Founder line 2 showed a strong and complete pattern of expression as compared to the other founders (data not shown), and was therefore used for all consecutive experiments.

### TdTomato is expressed in Prox1 positive cells upon crossing of the LSL-tdTomato reporter mice with a Prox1-Cre-ERT2 line

**Fig 3:**
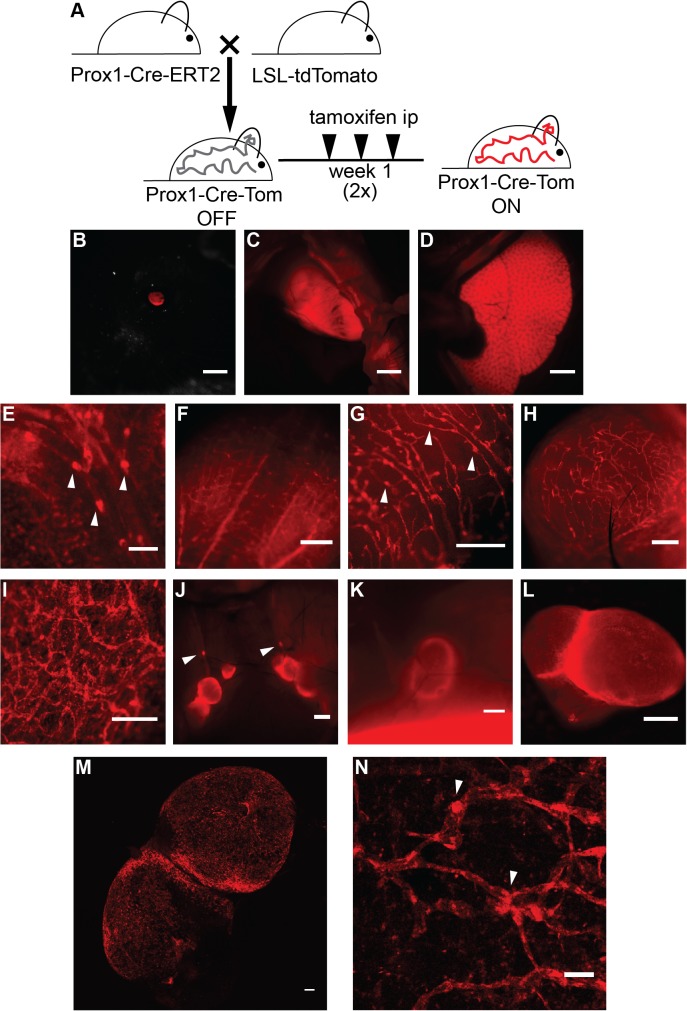
TdTomato is expressed in Prox1 positive cells upon crossing of the reporter mice with a Prox1-Cre-ERT2 line. **A** Schematic representation of the breeding to Prox1-Cre-ERT2 and the tamoxifen administration regimen utilized (1 μg/g body weight in sunflower seed oil, intraperitoneally (ip), three times a week for two weeks). TdTomato expression was analyzed with a stereomicroscope and was detected in the eye (**B**), heart (**C**) and liver (**D**). TdTomato was visible in lymphatic structures in the mesentery (**E**), tongue (**F**), uterus (**G**), bladder (**H**), ear skin (ripped in half, **I**), lymph nodes of the neck area (**J**) and the inguinal lymph node (**K**). Ex vivo imaging of an inguinal lymph node (**L**). **M** Confocal imaging of tdTomato autoflorescence showed lymphatic structures in a freshly isolated lymph node. Maximal intensity projection of a tile scan, z-stack of the lymph node is shown. **N** Confocal imaging (maximal intensity projection of a z-stack) of tdTomato autoflorescence showed lymphatic structures in a freshly isolated split ear sample. Arrowheads indicate lymphatic valves. Scale bars = 2000 μm (D-C), 1000 μm (J, K), 500 μm (E-I, L), 100 μm (M-N). Images shown are representative of 3 transgenic animals analyzed from 2 independent litters.

The transcription factor Prox1 regulates the development of the lymphatic system and maintains lymphatic identity throughout life [21, 22]. We crossed the previously characterized Prox1-Cre-ERT2 mouse line [12] with the LSL-tdTomato reporter line. In order to induce Cre expression, adult mice were injected intraperitoneally with tamoxifen (50 μg/g body weight), three times a week for two weeks and analyzed as early as two days after the last application (Fig 3A). Tamoxifen-treated mice were imaged using a stereomicroscope. Known Prox1 expressing organs, including the lens (Fig 3B), the heart (Fig 3C) and the liver (Fig 3D), showed strong fluorescence. LVs were clearly identifiable in different anatomical locations due to their clear tdTomato expression. Fig 3 shows representative stereomicroscopic images of LVs in the mesentery (E), tongue (F), uterus (G), bladder (H), inguinal lymph node (K, L), auricular lymph node (J) and ear skin (I). In many organs, lymphatic valves were readily visible and characterized by a stronger tdTomato fluorescence (arrowheads in Fig 3). Freshly isolated and unfixed lymph node and ear skin samples were also analyzed by confocal microscopy. Fig 3M shows clearly visible tdTomato endogenous fluorescence in the lymph node subcapsular sinus. In the ear skin (Fig 3N), endogenous tdTomato signal was found in LV structures, with a stronger signal in valves (arrowheads in Fig 3N). Some single cells positive for tdTomato were also visible. Taken together, these data show that tdTomato expression faithfully recapitulated Prox1 expression.

### TdTomato expression co-localizes with lymphatic markers in different organs

**Fig 4:**
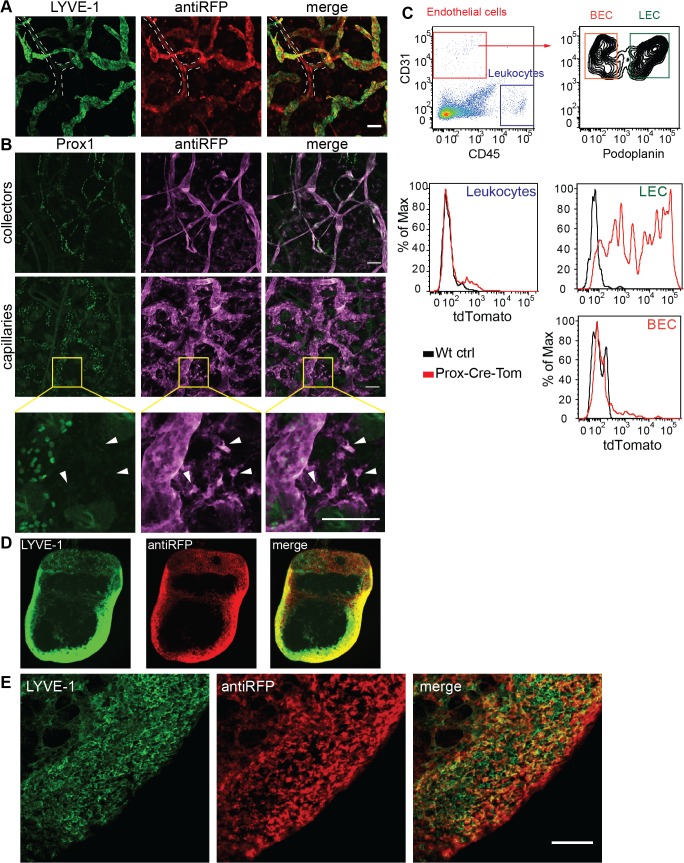
TdTomato expression co-localizes with lymphatic markers in different organs. **A** Whole mount preparations of ear samples showed co-localization of the lymphatic marker LYVE-1 with tdTomato (detected with an anti-RFP antibody). Dashed line indicates a lymphatic collector vessel (LYVE-1 low, tdTomato high). **B** Whole mount preparations of ear samples showed co-localization of the lymphatic marker Prox1 with tdTomato. Upper panels show lymphatic collectors, middle panels show lymphatic capillaries and lower panels show a magnification of the area indicated in the middle panels. **C** FACS single cell analyses revealed tdTomato expression in the LEC population (CD45- CD31+ podoplanin+), but not in the BEC (CD45- CD31+ podoplanin-) or in the leukocyte (CD45+) populations. **D** Ultramicroscopic analysis of an immunostained and optically cleared lymph node showed colocalization of LYVE-1 and tdTomato. Maximum projection of a 260 μm z-stack. **E** Whole mount preparation of a lymph node sample showed the presence of LYVE-1+RFP+ lymphatic endothelium in the subcapsular sinus. LYVE-1+RFP- single cells are most likely macrophages. Data are representative of 4 transgenic animals analyzed from 2 independent litters. Scale bars: 100 μm.

In order to confirm specific tdTomato expression in LVs, we performed whole mount immunofluorescent stainings of PFA fixed ear skin using an antibody against tdTomato (RFP), since the endogenous tdTomato signal could not be preserved after PFA fixation, and the established lymphatic markers Prox1 and LYVE-1. TdTomato co-localized with LYVE-1 positive lymphatic capillaries in the skin (Fig 4A). Moreover, lymphatic collectors, which are characterized by a lower expression of LYVE-1 but retain Prox1 expression in adult mice, were strongly positive for tdTomato (Fig 4A, dashed line). TdTomato positive lymphatic capillaries and collectors were also Prox1 positive (Fig 4B). In ear skin, some tdTomato single cells were also visible, but they did not co-localize with LYVE-1 or Prox1 (Fig 4A and 4B). These single cells were also negative for the major histocompatibility complex (MHC) II expressed by antigen presenting cells, and for the pan-leukocyte marker CD45 (not shown). FACS analysis of ear single cell suspension (Fig 4C) revealed that LECs (CD45- CD31+ podoplanin+) expressed tdTomato. In contrast, blood vascular endothelial cells (BEC, CD45- CD31+ podoplanin-) and leukocytes (CD45+) did not show any tdTomato expression. We additionally performed ultramicroscopic analysis of lymph nodes that were immunostained for LYVE-1 and tdTomato and then were optically cleared (Fig 4D). We found a clear co-localization of the lymphatic marker LYVE-1 with tdTomato (Fig 4D). The pattern of LVs in the lymph node was prominent in the subcapsular sinus and between the lobes. Further confocal analysis of lymph node whole-mount preparations showed the presence of LYVE-1+RFP+ LVs together with LYVE-1+RFP- cells, presumably macrophages, in the subcapsular sinus (Fig 4E). Collectively, these data confirm the lymphatic identity of the tdTomato-positive vessel-like structures.

### Comparison of the Prox1-Cre-tdTomato mouse with other RFP reporters

**Fig 5:**
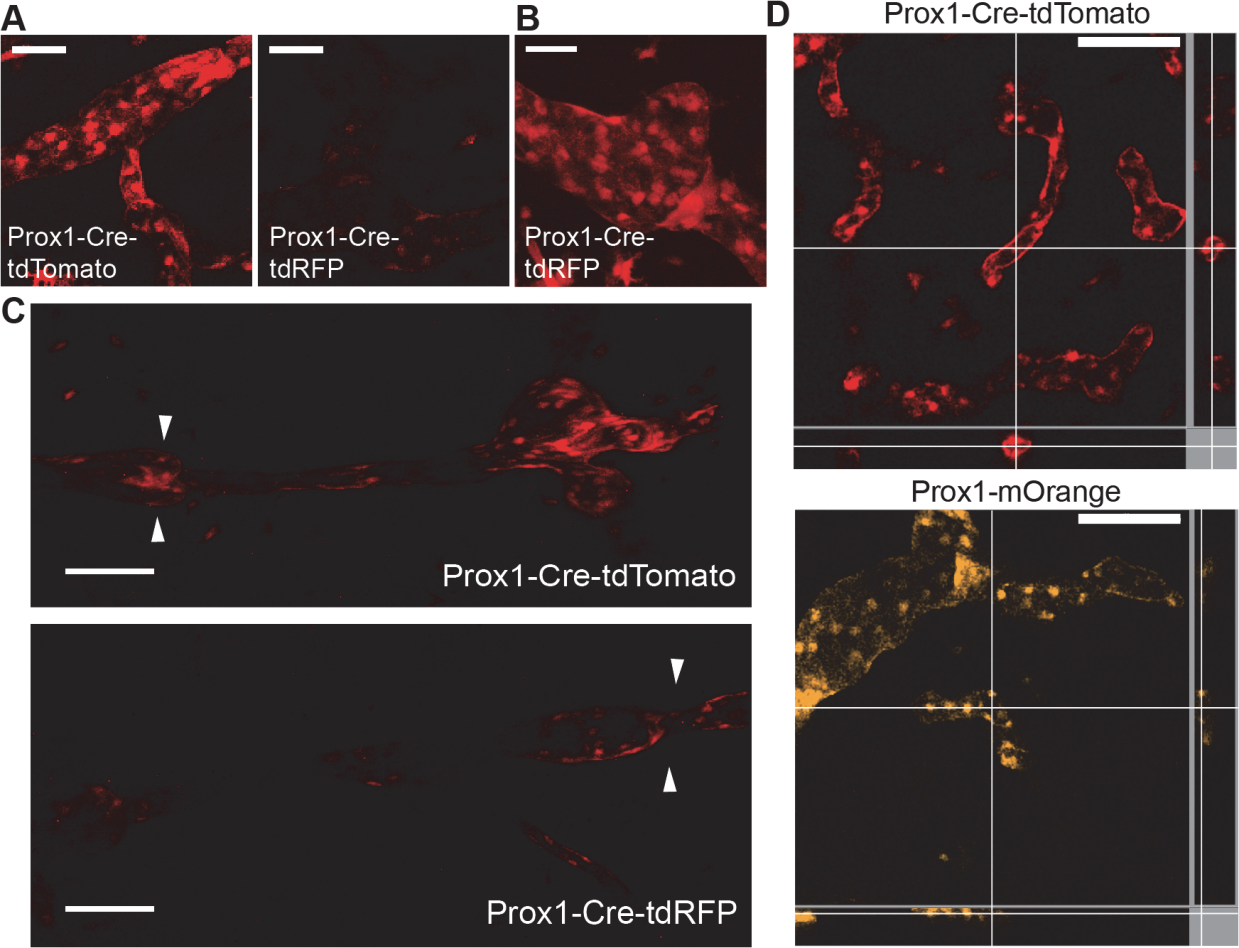
Comparison of the Prox1-Cre-tdTomato mouse with the Prox1-Cre-tdRFP mouse. **A** Intravital confocal microscopy revealed brighter lymphatic vessels in the Prox1-Cre-tdTomato mouse than in the Prox1-Cre-tdRFP mouse (images acquired applying the same laser power and settings). **B** LVs can be visualized in Prox1-Cre-tdRFP mice only by applying a higher laser power. **C** Intravital multiphoton microscopy allowed the imaging of deep lymphatic collectors (arrowheads indicate valves), which were better visualized in Prox1-Cre-tdTomato mice. **D** Orthogonal analysis showed better visualization of the lymphatic vessel lumen in Prox1-Cre-tdTomato mice than in Prox1-mOrange mice, suggesting diffuse dye distribution in LECs. Scale bars: 100 μm.

In order to compare the tdTomato reporter to other red fluorescent reporters for the imaging of LVs, we directly compared Prox1-Cre-tdTomato mice with Prox1-Cre-ERT2 mice crossed with the previously established reporter line tdRFP [16]. Intravital confocal microscopic analysis of mouse ear skin performed with the same microscope settings and laser power showed considerably brighter LVs in the Prox1-Cre-tdTomato mouse than in the Prox1-Cre-tdRFP mouse (Fig 5A). Only the application of a higher laser power allowed visualization of LVs in the Prox1-Cre-tdRFP mouse (Fig 5B). Multiphoton microscopy was used to reach deeper into the tissue and to image collecting vessels. Also in this setting, the Prox1-Cre-tdTomato mouse performed better than the Prox1-Cre-tdRFP mouse (Fig 5C).

Additionally, we imaged a previously characterized LV reporter line, namely the Prox1-mOrange mice which express the fluorescent protein mOrange under direct Prox1 transcriptional control [6]. Intravital confocal imaging of ear skin showed clear labeling of LVs (Fig 5D). However, the distribution of the fluorescent signal appeared more nuclear as compared to the Prox1-Cre-tdTomato mouse, as shown in the orthogonal view where the lumen of the LVs was better visualized (Fig 5D).

### The Prox1-Cre-tdTomato mouse is a valid tool for intravital microscopy of DC migration

**Fig 6:**
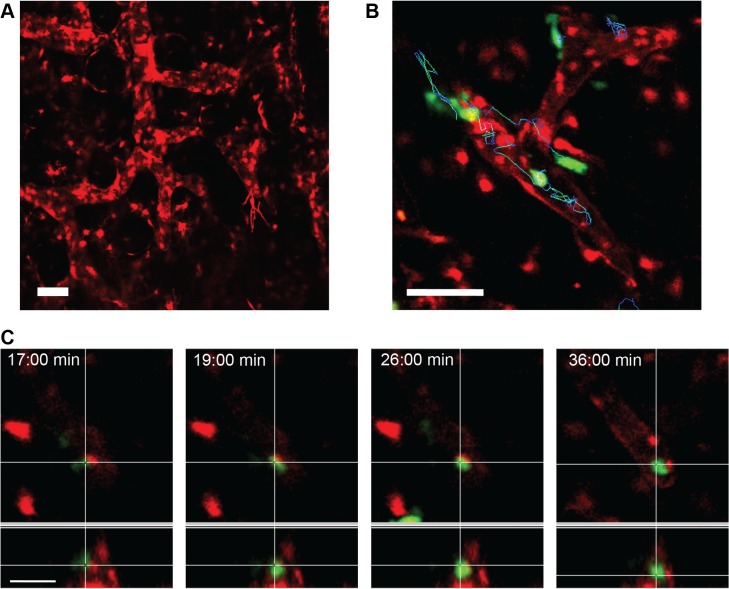
The Prox1-Cre-tdTomato mouse is a valid tool for intravital microscopy of DC migration. **A** Maximum intensity projection of a z-stack of a mouse ear imaged with the IVM settings. The tdTomato positive LVs were clearly visible. Scale bar: 70 μm. **B** YFP-DCs were injected into the ear and imaged for 1 hour in an inverted confocal microscope. The image corresponds to the maximum intensity projection of single frame z-stack where the migratory tracks of DCs are shown. Scale bar: 70 μm. **C** Orthogonal view of the entry of a DC into the LV is visualized over time. Four representative time points from a 1-hour video are shown. Scale bar: 20 μm.

One of the major functions of the lymphatic system is the trafficking of DCs from the periphery to the lymph nodes [23]. To test the use of the lymphatic reporter mouse for the analysis of DC migration, Prox1-Cre-tdTomato mice were injected with YFP-expressing DCs and imaged by intravital microscopy (IVM). Endogenous tdTomato-fluorescence was clearly visible in the LVs imaged with the IVM settings (Fig 6A). YFP-DCs were imaged for 1 hour and could be easily tracked inside the LVs or in the interstitium (S1 Video). Fig 6B shows the tracks of several DC crawling inside LVs. It was possible to image *in vivo* over time the entry of DC into the lymphatic capillaries. As shown in the representative orthotopic view (Fig 6C) and in the S2 Video, an YFP-positive DC makes first contact with a tdTomato-positive LV and finally enters it, squeezing through the cell-cell openings. These data show the applicability of the Prox1-Cre-tdTomato mouse to the analysis of immune cell trafficking into and within the LVs *in vivo*.

### The Prox1-Cre-tdTomato mouse facilitates the FACS sorting of LECs

**Fig 7:**
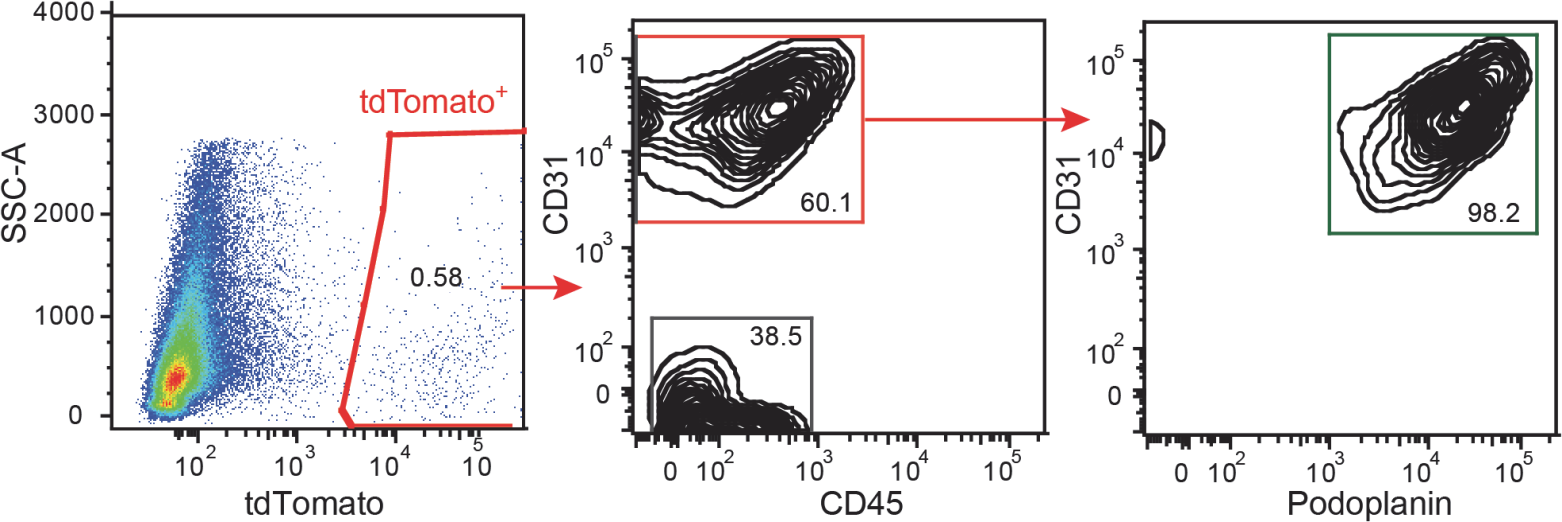
The Prox1-Cre-tdTomato mouse facilitates FACS sorting of LECs. An ear single cell suspension was analyzed with a BD Fortessa flow cytometer. The tdTomato+ population contained 60% of CD45- CD31+ podoplanin+ LEC and 40% of CD45- CD31- cells. The sorting of tdTomato+ CD31+ cells allowed the isolation of a pure LEC population. Data are representative of 4 transgenic animals analyzed from 2 independent litters.

LECs finely regulate their gene expression in response to inflammatory stimuli and play an important role in modulating inflammatory responses, as shown by gene expression analyses of sorted LEC from inflamed and uninflamed tissues [4, 24]. Until now, staining for multiple antigens is needed in order to sort pure populations of LECs from complex single cell suspensions [25]. We next investigated whether the Prox1-Cre-tdTomato mice might simplify LEC isolation from ear single cell suspensions. We found that about 60% of tdTomato-positive cells were CD45-negative, CD31-positive endothelial cells (Fig 7). An additional CD45-negative, CD31-negative population accounted for about 40% of tdTomato-positive events (Fig 7). Among the endothelial cells, 98% were identified as LEC (CD31-positive and podoplanin-positive, Fig 7), suggesting that a pure LEC population can be obtained from the ears of Prox1-Cre-tdTomato mice by sorting tdTomato-positive, CD31-positive cells, simplifying existing sorting protocols [25]. The identity of the additional tdTomato-positive population remains to be elucidated.

### Progressive enlargement of the lymphatic vessel diameter during the early phases of skin inflammation

**Fig 8:**
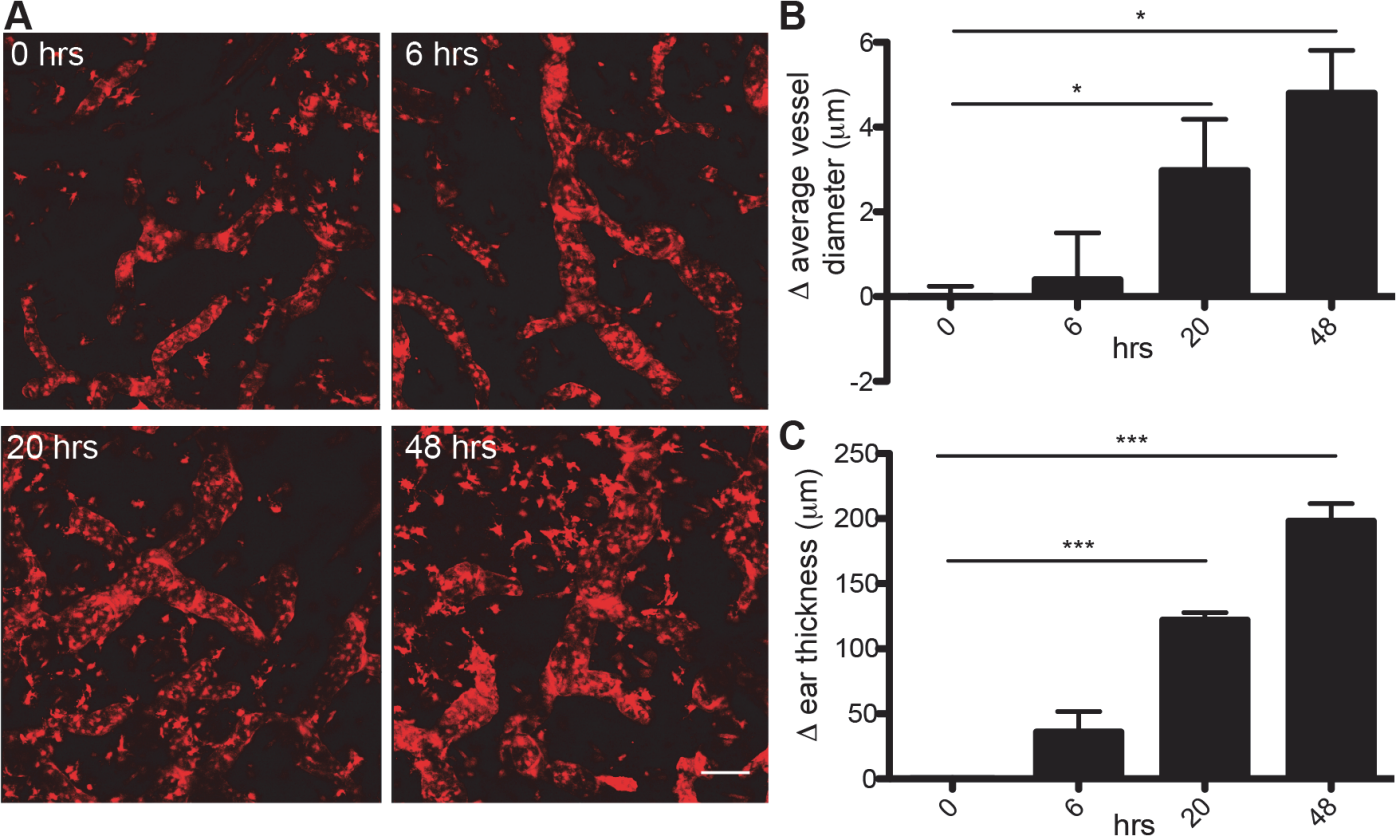
Progressive enlargement of the lymphatic vessel diameter during the early phases of skin inflammation. **A** Prox1-Cre-tdTomato mice (n = 5) were imaged at 0, 6, 20 and 48 hrs after the induction of a cutaneous hypersensitivity reaction by intravital confocal microscopy. Scale bars: 100 μm. **B** Morphological analysis revealed progressive enlargement of the LV diameter over time. Data represent means ± SEM, statistical significance was analyzed using the unpaired student t-test, *p<0.05. **C** Ear thickness increased over time. Data represent means ± SEM, statistical significance was analyzed using 1-way ANOVA and Dunnett’s post test, ***p<0.0001.

We next used the Prox1-Cre-tdTomato mouse model to investigate the early morphological changes that the lymphatic vasculature might undergo upon induction of inflammation. To this aim, we induced a contact hypersensitivity reaction to oxazolone in the ears and performed intravital confocal microscopy over time (Fig 8A). We observed a progressive enlargement of the LV diameter during the first 48 hours after induction of inflammation (Fig 8B). However, the total vessel length did not change during the time points analyzed (not shown). Together with the LV enlargement, the ear thickness progressively increased (Fig 8C). Collectively, these observations indicate that enlargement of the vessel diameter represents the earliest morphological change that occurs in LVs upon acute inflammation.

## Discussion

In this report, we describe the generation of an inducible tdTomato reporter mouse and its applicability to lymphatic research. The induced Prox1-Cre-tdTomato mice showed bright red fluorescence in LVs and could be successfully applied to *in vivo* microscopy of DC migration and of LV morphology during acute inflammation, and to FACS analyses of LECs isolated from complex tissues.

TdTomato is the brightest among the red-fluorescent proteins, derived from directed evolution of DsRed [26], and it is therefore well-suited for *in vivo* imaging and multi-color FACS analysis. Accordingly, we found that the tdTomato reporter mouse had increased brightness of LVs and allowed an increased depth of imaging, as compared to a previously generated tdRFP reporter line [16]. Moreover, tdTomato is more photostable than tdRFP since we observed considerably less photobleaching when performing IVM of DC migration over one hour in Prox1-Cre-tdTomato mice as compared to our previous experience with VE-cadherin-Cre-tdRFP mice [18].

We cloned tdTomato under the control of a strong ubiquitous promoter and a transcriptional/translational-floxed STOP cassette, and generated a transgenic reporter line. In a preliminary study, we crossed the obtained founder lines to an inducible, skin specific, Cre recombinase expressing mouse line: K5-Cre-ERT2. Surprisingly, founder line 2, which was characterized by the least number of copies, had the strongest and most uniform tdTomato expression. Since the reporter was generated by pronuclear microinjection, which results in random transgene integration into the genome, we assume the impact of position effects on the expression pattern. It is possible that the integration sites of founders with higher copy numbers are in genomic regions that disturbed their transcription, or the high copy number itself was responsible for repeat-induced silencing of the transgene [27, 28].

In contrast to the previously published BAC-based models for fluorescent imaging of lymphatic vessels [5–7], our approach involved the generation of a reporter mouse with a floxed STOP cassette followed by tdTomato, and its crossing with an inducible, lymphatic specific Cre expressing line, allowing not only tissue specificity, but also time specificity of induction. Several lymphatic specific Cre lines have been generated so far. Most of them, however, are characterized by extra-lymphatic Cre expression. A LYVE-1-Cre line expressed Cre recombinase in a subset of blood vascular endothelial cells and leukocytes [29]. A podoplanin-Cre line was also described [30], but the expression of this transmembrane protein is also prominent in stromal cells of lymph nodes and secondary lymphoid organs; therefore, it is not well suited for LV imaging and analysis in the lymph node. Thus, we chose to cross the reporter line to a previously described Prox1-Cre-ERT2 line [12]. This line shows some extra-lymphatic expression of Cre recombinase, in line with the known function of Prox1 as a transcription factor in specific tissues. We detected strong tdTomato expression in the liver, heart and lens, where Prox1 is normally expressed [5, 7]. Importantly, in anatomical locations where LVs are usually analyzed, such as the skin and the lymph nodes, tdTomato expression was strong on LVs, as evidenced by co-staining for the lymphatic markers LYVE-1, Prox1 and podoplanin in whole-mount preparations and FACS analyses. It is of interest that a yet unknown subpopulation of single cells positive for tdTomato was identified in the skin. As shown by whole-mount stains and FACS analyses, this population was not positive for Prox1, MHC-II or CD45. Characterization of the previously described Prox1-driven lymphatic reporters, namely the ProxTom [7] and the Prox1-GFP mouse lines [5], did not indicate the presence of this single cell population in the skin. The identity of this population is presently not clear and merits further study. Nevertheless, since this cell population appeared to be immobile, its presence did not interfere with the IVM analysis of DC migration.

The direct fluorescent visualization of the lymphatic vasculature in distinct transgenic and knockout mouse models could be advantageous. Since the Prox1-Cre-tdTomato mouse features two alleles transmitted independently (the Prox1-driven Cre recombinase and the inducible tdTomato reporter), this application will require the generation of triple-transgenic mice.

Tissue fixation with PFA required the use of an anti-RFP antibody to detect tdTomato, since the endogenous fluorescence could not be preserved. Other fixation methods such as periodate-lysine-paraformaldehyde might preserve endogenous tissue fluorescence, as described by Truman et al [7]. Moreover, whole mount staining for some antigens might also work omitting the fixation step. However, in many applications, such as IVM, FACS analysis and stereomicroscopic analysis, tissue fixation is not required and the endogenous fluorescence could be easily detected.

Application of the Prox1-Cre-tdTomato mouse for the analysis of DC migration in the ear skin enabled the observation of DCs interacting with LECs and their entry into the LVs. Once inside the vessels, DCs actively migrated and crawled within the LVs. Until recently, DCs were thought to be passively transported by flow from the periphery to the draining lymph nodes once they had entered the LV. Recent studies, however, have shown that after transmigration, DCs actively crawl within the LV [18, 31]. DC migration has been shown to be integrin-independent [32], whereas the CCR7-CCL21 axis plays a key function [31]. In these reports, the visualization of the lymphatic vasculature was obtained either by antibody staining [31, 32] or by the use of a pan-vascular reporter mouse, the VE-cadherin-Cre mouse line, in which both blood and lymphatic vessels can be visualized [18]. Our model has some advantages compared to the latter ones, since it can specifically visualize LVs and does not require antibody staining. Moreover, the LVs in our model are red-fluorescent and enable the combined visualization of GFP and YFP fluorescent leukocytes. This model could therefore be applied to further IVM studies, aiming to unravel additional cellular players and molecular interactions involved in the complex mechanisms of DC migration.

We also applied our animal model to the simplification of existent LEC FACS sorting protocols [25]. Single-cell sorting is a powerful tool to isolate a pure cell population from a complex tissue digest and to analyze its protein or gene expression. This technique allowed not only the discovery of novel molecules differentially expressed by LECs and BECs [33], but also the investigation of the changes in gene expression that occur in LECs upon different inflammatory stimuli [34]. Our animal model allowed the separation of a pure LEC population from ear single cell suspensions by use of the intrinsic tdTomato fluorescence and staining for the pan-endothelial marker CD31, thereby simplifying the 4-colour staining currently used. Our data are in agreement with a previous report that took advantage of the ProxTom mouse [35].

Finally, the Prox1-Cre-tdTomato mouse allowed to directly visualize and monitor over time by IVM the first morphological changes of LVs that occur during acute inflammation, namely the progressive enlargement of LVs diameter, using a cutaneous contact hypersensitivity model. Until now, most related studies have focused on later time points of inflammation and have analyzed morphological changes in the lymphatic vasculature by histology [17]. The possibility to image the same mouse at different time points also provides the advantage to reduce the number of animals needed for analysis.

Collectively, our data show that the Prox1-Cre-tdTomato mouse model shows bright red-fluorescent LVs and has important applications to IVM of leukocyte migration into and within LVs, *in vivo* studies of LV morphology and *ex vivo* FACS analyses of LEC. It is therefore a useful novel tool for the study of LVs in physiological and pathological conditions and it will be certainly of great use to lymphatic research.

## Supporting Information

S1 Video Intravital microscopy of dendritic cell migration. YFP-DCs were injected into the ear and a frame was acquired every thirty seconds for 1 hour in an inverted confocal microscope. The video shows DCs (green) moving in the interstitium and in lymphatic vessels (red). Video speed: 5 frames per second.
(AVI)

S2 Video Intravital microscopy of dendritic cell migration. YFP-DCs were injected into the ear and a frame was acquired every thirty seconds for 1 hour in an inverted confocal microscope. The video shows a DC (green) entering a lymphatic vessel (red). An orthogonal view is provided to enhance visibility of the DC location inside the lymphatic vessel. Video speed: 5 frames per second.
(AVI)

## References

[1] Jurisic G, Detmar M. Lymphatic endothelium in health and disease. Cell Tissue Res. 2009; 335(1): 97–108. 10.1007/s00441-008-0644-2 18648856

[2] Alitalo K. The lymphatic vasculature in disease. Nat Med. 2011; 17(11): 1371–80. 10.1038/nm.2545 22064427

[3] Alitalo A, Detmar M. Interaction of tumor cells and lymphatic vessels in cancer progression. Oncogene. 2012; 31(42): 4499–508. 10.1038/onc.2011.602 22179834

[4] Dieterich LC, Seidel CD, Detmar M. Lymphatic vessels: new targets for the treatment of inflammatory diseases. Angiogenesis. 2014; 17(2): 359–71. 10.1007/s10456-013-9406-1 24212981

[5] Choi I, Chung HK, Ramu S, Lee HN, Kim KE, Lee S, et al Visualization of lymphatic vessels by Prox1-promoter directed GFP reporter in a bacterial artificial chromosome-based transgenic mouse. Blood. 2011; 117(1): 362–5. 10.1182/blood-2010-07-298562 20962325 PMC3037757

[6] Hagerling R, Pollmann C, Kremer L, Andresen V, Kiefer F. Intravital two-photon microscopy of lymphatic vessel development and function using a transgenic Prox1 promoter-directed mOrange2 reporter mouse. Biochem Soc Trans. 2011; 39(6): 1674–81. 10.1042/BST20110722 22103506

[7] Truman LA, Bentley KL, Smith EC, Massaro SA, Gonzalez DG, Haberman AM, et al ProxTom lymphatic vessel reporter mice reveal Prox1 expression in the adrenal medulla, megakaryocytes, and platelets. Am J Pathol. 2012; 180(4): 1715–25. 10.1016/j.ajpath.2011.12.026 22310467 PMC3349900

[8] Martinez-Corral I, Olmeda D, Dieguez-Hurtado R, Tammela T, Alitalo K, Ortega S. In vivo imaging of lymphatic vessels in development, wound healing, inflammation, and tumor metastasis. Proc Natl Acad Sci U S A. 2012; 109(16): 6223–8. 10.1073/pnas.1115542109 22474390 PMC3341065

[9] Mumprecht V, Honer M, Vigl B, Proulx ST, Trachsel E, Kaspar M, et al In vivo imaging of inflammation- and tumor-induced lymph node lymphangiogenesis by immuno-positron emission tomography. Cancer Res. 2010; 70(21): 8842–51. 10.1158/0008-5472.CAN-10-0896 20978206 PMC3398152

[10] Proulx ST, Luciani P, Derzsi S, Rinderknecht M, Mumprecht V, Leroux JC, et al Quantitative imaging of lymphatic function with liposomal indocyanine green. Cancer Res. 2010; 70(18): 7053–62. 10.1158/0008-5472.CAN-10-0271 20823159 PMC3398157

[11] Proulx ST, Luciani P, Christiansen A, Karaman S, Blum KS, Rinderknecht M, et al Use of a PEG-conjugated bright near-infrared dye for functional imaging of rerouting of tumor lymphatic drainage after sentinel lymph node metastasis. Biomaterials. 2013; 34(21): 5128–37. 10.1016/j.biomaterials.2013.03.034 23566803 PMC3646951

[12] Bazigou E, Lyons OT, Smith A, Venn GE, Cope C, Brown NA, et al Genes regulating lymphangiogenesis control venous valve formation and maintenance in mice. J Clin Invest. 2011; 121(8): 2984–92. 10.1172/JCI58050 21765212 PMC3223924

[13] Sawicki JA, Morris RJ, Monks B, Sakai K, Miyazaki J. A composite CMV-IE enhancer/beta-actin promoter is ubiquitously expressed in mouse cutaneous epithelium. Exp Cell Res. 1998; 244(1): 367–9. 9770380 10.1006/excr.1998.4175

[14] Buchholz F, Angrand PO, Stewart AF. A simple assay to determine the functionality of Cre or FLP recombination targets in genomic manipulation constructs. Nucleic Acids Res. 1996; 24(15): 3118–9. 8760904 10.1093/nar/24.15.3118 PMC146033

[15] Kataoka K, Kim DJ, Carbajal S, Clifford JL, DiGiovanni J. Stage-specific disruption of Stat3 demonstrates a direct requirement during both the initiation and promotion stages of mouse skin tumorigenesis. Carcinogenesis. 2008; 29(6): 1108–14. 10.1093/carcin/bgn061 18453544 PMC2902397

[16] Luche H, Weber O, Nageswara Rao T, Blum C, Fehling HJ. Faithful activation of an extra-bright red fluorescent protein in "knock-in" Cre-reporter mice ideally suited for lineage tracing studies. Eur J Immunol. 2007; 37(1): 43–53. 17171761 10.1002/eji.200636745

[17] Kunstfeld R, Hirakawa S, Hong YK, Schacht V, Lange-Asschenfeldt B, Velasco P, et al Induction of cutaneous delayed-type hypersensitivity reactions in VEGF-A transgenic mice results in chronic skin inflammation associated with persistent lymphatic hyperplasia. Blood. 2004; 104(4): 1048–57. 15100155 10.1182/blood-2003-08-2964

[18] Nitschke M, Aebischer D, Abadier M, Haener S, Lucic M, Vigl B, et al Differential requirement for ROCK in dendritic cell migration within lymphatic capillaries in steady-state and inflammation. Blood. 2012; 120(11): 2249–58. 10.1182/blood-2012-03-417923 22855606

[19] Halin C, Tobler NE, Vigl B, Brown LF, Detmar M. VEGF-A produced by chronically inflamed tissue induces lymphangiogenesis in draining lymph nodes. Blood. 2007; 110(9): 3158–67. 17625067 10.1182/blood-2007-01-066811 PMC2200913

[20] Hagerling R, Pollmann C, Andreas M, Schmidt C, Nurmi H, Adams RH, et al A novel multistep mechanism for initial lymphangiogenesis in mouse embryos based on ultramicroscopy. EMBO J. 2013; 32(5): 629–44. 10.1038/emboj.2012.340 23299940 PMC3590982

[21] Wigle JT, Oliver G. Prox1 function is required for the development of the murine lymphatic system. Cell. 1999; 98(6): 769–78. 10499794 10.1016/s0092-8674(00)81511-1

[22] Hong YK, Harvey N, Noh YH, Schacht V, Hirakawa S, Detmar M, et al Prox1 is a master control gene in the program specifying lymphatic endothelial cell fate. Dev Dyn. 2002; 225(3): 351–7. 12412020 10.1002/dvdy.10163

[23] Forster R, Braun A, Worbs T. Lymph node homing of T cells and dendritic cells via afferent lymphatics. Trends Immunol. 2012; 33(6): 271–80. 10.1016/j.it.2012.02.007 22459312

[24] Aebischer D, Iolyeva M, Halin C. The inflammatory response of lymphatic endothelium. Angiogenesis. 2014; 17(2): 383–93. 10.1007/s10456-013-9404-3 24154862

[25] Halin C, Detmar M. Chapter 1. Inflammation, angiogenesis, and lymphangiogenesis. Methods Enzymol. 2008; 445: 1–25. 10.1016/S0076-6879(08)03001-2 19022053

[26] Shaner NC, Campbell RE, Steinbach PA, Giepmans BN, Palmer AE, Tsien RY. Improved monomeric red, orange and yellow fluorescent proteins derived from Discosoma sp. red fluorescent protein. Nat Biotechnol. 2004; 22(12): 1567–72. 15558047 10.1038/nbt1037

[27] Clark AJ, Bissinger P, Bullock DW, Damak S, Wallace R, Whitelaw CB, et al Chromosomal position effects and the modulation of transgene expression. Reprod Fertil Dev. 1994; 6(5): 589–98. 7569038 10.1071/rd9940589

[28] Garrick D, Fiering S, Martin DI, Whitelaw E. Repeat-induced gene silencing in mammals. Nat Genet. 1998; 18(1): 56–9. 9425901 10.1038/ng0198-56

[29] Pham TH, Baluk P, Xu Y, Grigorova I, Bankovich AJ, Pappu R, et al Lymphatic endothelial cell sphingosine kinase activity is required for lymphocyte egress and lymphatic patterning. J Exp Med. 2009; 207(1): 17–27. 10.1084/jem.20091619 20026661 PMC2812554

[30] Onder L, Scandella E, Chai Q, Firner S, Mayer CT, Sparwasser T, et al A novel bacterial artificial chromosome-transgenic podoplanin-cre mouse targets lymphoid organ stromal cells in vivo. Front Immunol. 2011; 2: 50 10.3389/fimmu.2011.00050 22566840 PMC3342134

[31] Tal O, Lim HY, Gurevich I, Milo I, Shipony Z, Ng LG, et al DC mobilization from the skin requires docking to immobilized CCL21 on lymphatic endothelium and intralymphatic crawling. J Exp Med. 2011; 208(10): 2141–53. 10.1084/jem.20102392 21930767 PMC3182054

[32] Lammermann T, Bader BL, Monkley SJ, Worbs T, Wedlich-Soldner R, Hirsch K, et al Rapid leukocyte migration by integrin-independent flowing and squeezing. Nature. 2008; 453(7191): 51–5. 10.1038/nature06887 18451854

[33] Jurisic G, Maby-El Hajjami H, Karaman S, Ochsenbein AM, Alitalo A, Siddiqui SS, et al An unexpected role of semaphorin3a-neuropilin-1 signaling in lymphatic vessel maturation and valve formation. Circ Res. 2012; 111(4): 426–36. 10.1161/CIRCRESAHA.112.269399 22723300 PMC3572231

[34] Vigl B, Aebischer D, Nitschké M, Iolyeva M, Röthlin T, Antsiferova O, et al Tissue inflammation modulates gene expression of lymphatic endothelial cells and dendritic cell migration in a stimulus-dependent manner. Blood. 2011; 118(1): 205–15. 10.1182/blood-2010-12-326447 21596851

[35] Truman LA, N AG, Bentley KL, Ruddle NH. Lymphatic vessel function in head and neck inflammation. Lymphat Res Biol. 2013; 11(3): 187–92. 10.1089/lrb.2013.0013 24044758 PMC3780307

